# Does PD-1 blockade play a decisive role in the pathological complete remission of unresectable MSS, BRAF V600E-mutated metastatic colorectal cancer: A case report

**DOI:** 10.3389/fonc.2022.976622

**Published:** 2023-01-13

**Authors:** Li Tan, Qing-Shu Li, Dong Peng, Yong Cheng

**Affiliations:** ^1^ Department of Gastrointestinal Surgery, The First Affiliated Hospital of Chongqing Medical University, Chongqing, China; ^2^ Department of Pathology, School of Basic Medicine, Chongqing Medical University, Chongqing, China

**Keywords:** colorectal cancer (CRC), programmed death-1 inhibitor (PD-1 inhibitor), immunotherapy, targeted therapy, microsatellite-stable (MSS), case report

## Abstract

**Background:**

Colorectal cancer (CRC) ranks third in highest incidence among human cancers. With the continuous development of anti-cancer drugs, CRC patients are treated more and more effectively. However, the treatment of patients with unresectable metastatic CRC (mCRC) remains a core point for surgeons worldwide, especially for those with microsatellite stability (MSS) and BRAF V600E mutation, who have been reported to have the worst prognosis.

**Case description:**

We report a case of pathological complete remission in a patient with unresectable MSS, BRAF V600E-mutated metastatic rectal cancer after using Vemurafenib and Cetuximab in combination with Camrelizumab.

**Conclusions:**

This case suggested that Vemurafenib and Cetuximab combined with Camrelizumab is effective in the treatment of MSS, BRAF V600E-mutated mCRC. To benefit more patients, further studies need to be completed.

## Introduction

Colorectal cancer (CRC) ranks third in highest incidence and is the second leading cause of death among human cancers according to GLOBOCAN 2020 (1). The absence of early clinical manifestations usually leads to advanced stage of the patient at the time of diagnosis. With the continuous development of anti-cancer drugs, CRC patients are treated more and more effectively. However, the good prognosis still heavily relies on radical surgery (2, 3). In recent years, targeted therapy and immunotherapy are showing a promising effect for unresectable advanced CRC patients; however, the indication is narrow, which depends on the results of genetic tests.

As a regulator of the mitogen-activated protein kinase (MAPK)/extracellular signal-regulated kinase (ERK) signaling pathway, BRAF takes part in cell division, differentiation, and secretion. V600E represents the most common alteration of the BRAF locus. Previous literature reported that BRAF has the highest mutation rate in melanoma (40%–60%) and hairy cell leukemia (about 100%) and is rarer in CRC at only 5%–15% (4). In China, the BRAF mutation rate in CRC is 5.5%, in which V600E is 3.3% (5).

According to mismatch gene expression, CRC can be classified into mismatch repair-deficient (dMMR)/microsatellite instability high (MSI-H) and mismatch repair-proficient (pMMR)/microsatellite stability (MSS). A meta-analysis showed that patients with MSI-H and BRAF wild type (WT) had the best prognosis, while patients with MSS and BRAF V600E have the worst prognosis (6).

A recent clinical trial showed that BRAF V600E inhibitor Encorafenib and EGFR antibody Cetuximab combined with PD-1 inhibitor Nivolumab are safe and sensitive in patients with MSS, BRAF V600E-mutated CRC. Among the 22 patients, objective response rate (ORR) was 50% and disease control rate (DCR) was 96% (7). However, none of the patients achieved complete remission. Here, we reported for the first time the use of the BRAF V600E inhibitor Vemurafenib and EGFR antibody Cetuximab in combination with PD-1 inhibitor Camrelizumab in a patient with unresectable MSS BRAF V600E-mutated mCRC, culminating in pathological complete remission.

## Case presentation

**Figure 1:**
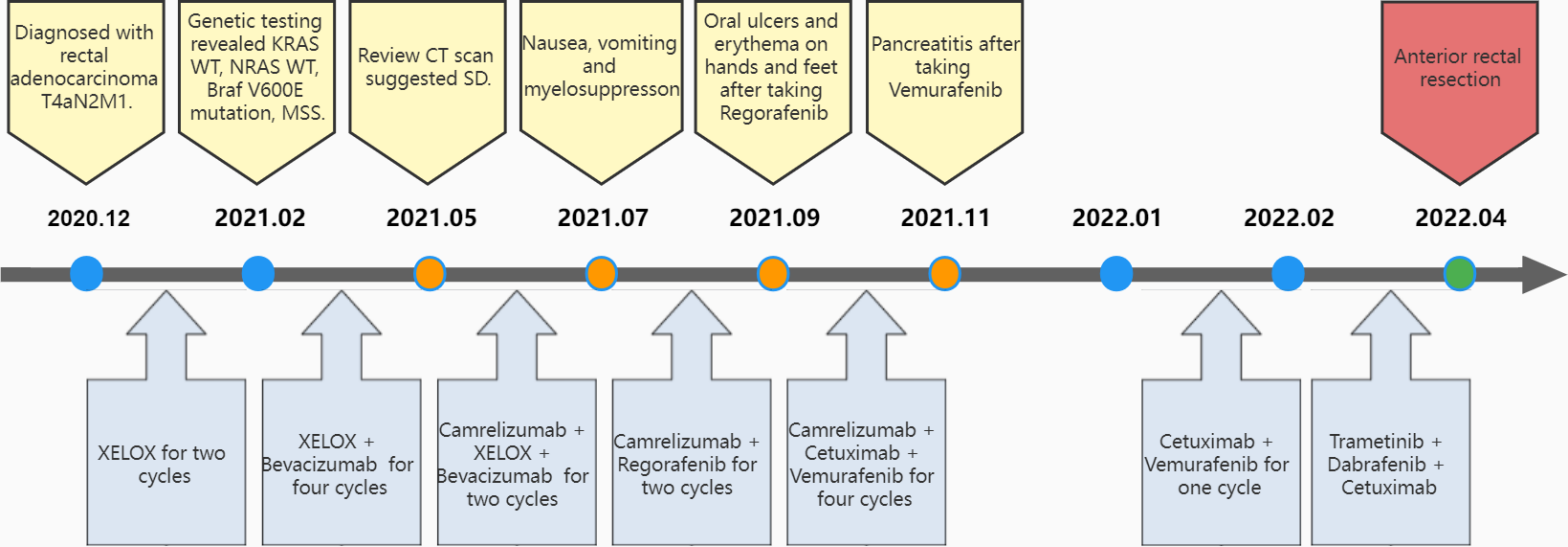
Flow of diagnosis and treatment. CT, computed tomography; SD, stable disease; XELOX, Capecitabine + Oxaliplatin regimen.

**Figure 2:**
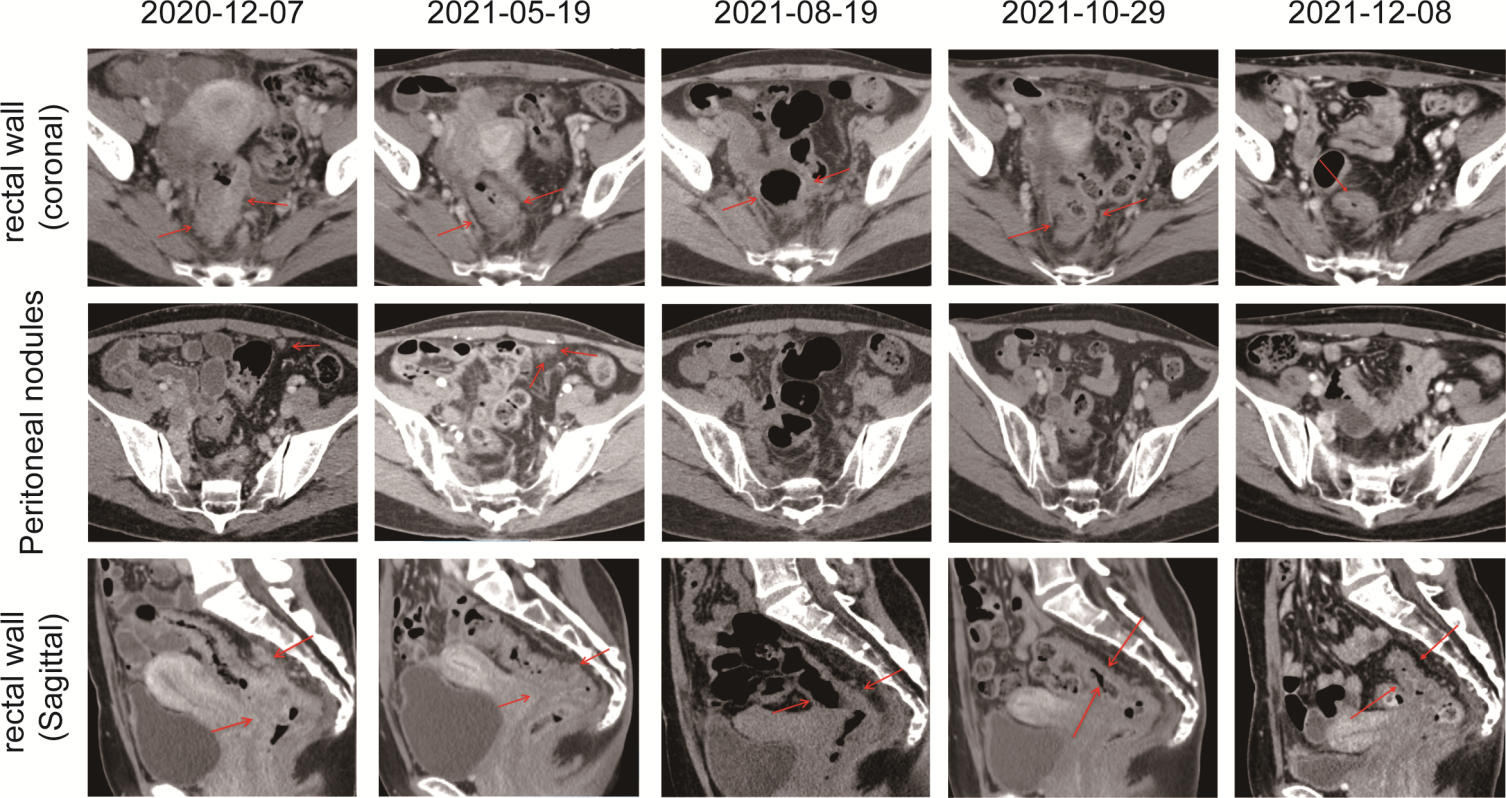
CT scans showed the chronological response of the patient following a series of treatment. The red arrow represents the area of the target lesion.

**Figure 3:**
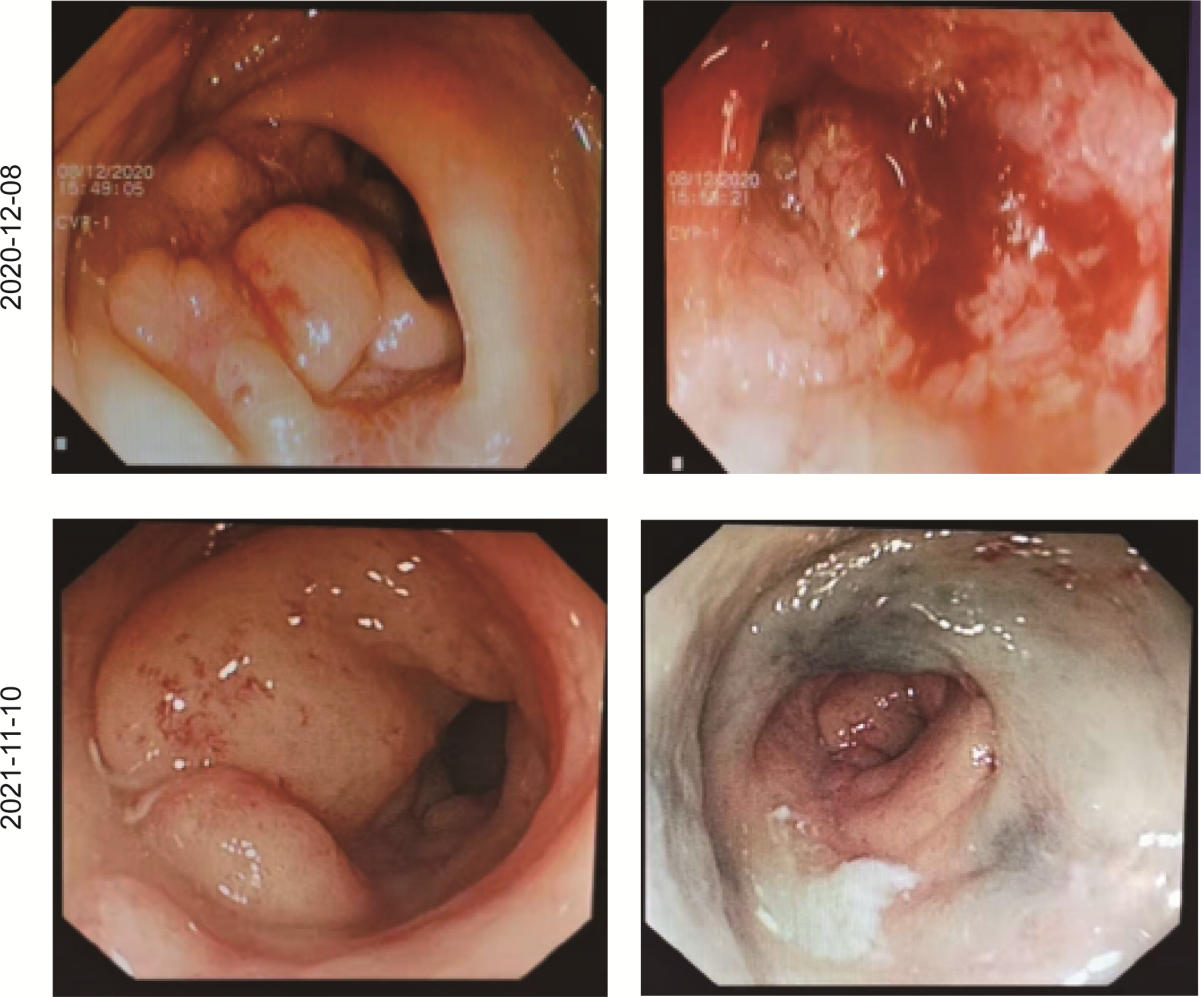
Coloscopy revealed the shrinking of the tumor following a series of treatments.

In December 2020, a 31-year-old woman was admitted to the First Affiliated Hospital of Chongqing Medical University. She had a change in stool pattern and habit for 8 months and anal pain with bloody stools for 6 months (**Figure 1**). Anal finger examination suggested a neoplasm in the anterior rectal wall about 5 cm from the anal verge. The size of the neoplasm was about 3 × 4 cm, invading half a circle of the intestinal wall, with unclear border, hard texture, poor mobility, no pressure pain, and no fluctuating feeling. No blood stain was found in the receding finger sleeve after examination. At this moment, computed tomography (CT) scan suggested extensive inhomogeneous thickening of the rectal wall with enhancement, considering malignant neoplastic lesions, and possible rectal cancer. The lesion broke through the serosa, with the surrounding fat gap blurred and the peritoneum, omentum, multiple irregular soft tissue nodules around the superior rectal artery, and presacral fascia multiple nodular enhanced; thus, the possibility of metastasis was considered (**Figure 2**). The colonoscopy suggested a new invasion of the intestine 5–13 cm from the anal verge, with localized erosion and necrosis (**Figure 3**). Biopsy suggested rectal adenocarcinoma. Magnetic resonance imaging (MRI) suggested that the lower margin of the tumor was about 7.5 cm from the anal verge, which was consistent with the manifestation of rectal cancer. According to the 2020 version of Chinese Society of Clinical Oncology (CSCO) guidelines for colorectal cancer, the MR stage was T4aN2, CRM (+), and EMVI (+). Positron emission computed tomography (PET-CT) suggested extensive heterogeneous thickening of the rectal wall, with unclear demarcation from the adjacent uterine wall and increased metabolic activity, which was consistent with the manifestation of rectal cancer. Multiple lymph node shadowing on the left side of the abdominal aorta, left parietal iliac vessels, right iliac fossa, and pelvic mesentery with partially increased metabolic activity was considered as metastasis (**Supplementary Figure 1**). The carcinoembryonic antigen (CEA) was 23 and carbohydrate antigen 19-9 (CA19-9) was 337.1 (**Supplementary Figure 2**). According to the 2020 version of CSCO guidelines for colorectal cancer, she was diagnosed with rectal adenocarcinoma T4aN2M1. There was no past medical history except that she had undergone cesarean section 10 years ago.

With no surgical opportunity, she received two cycles of chemotherapy with the XELOX regimen from December 2020 to January 2021. Then, on account of genetic testing that revealed KRAS WT, NRAS WT, BRAF V600E mutation, MSS, and high tumor mutation burden (TMB) (9.75 mutations/Mb) (**Supplementary Figure 3**), the chemotherapy regimen was changed to XELOX + Bevacizumab for four cycles, and radiotherapy (CTV 50Gy/25F, PTV 50Gy/25F) was administered simultaneously from February 2021 to May 2021. In 26/04/2021, the CEA decreased to 8 and CA19-9 decreased to 125.5, suggesting that the therapy was effective. However, when reviewing the CT scan on 19/5/2021, multiple nodules occurred in rectal lesions, superior rectal artery, presacral fascia, peritoneum, and omentum, which were similar to the previous case. CEA was elevated to 10 and CA 19-9 was elevated to 166.9. This suggested progressive disease, and it was proposed to change the treatment plan.

**Figure 4:**
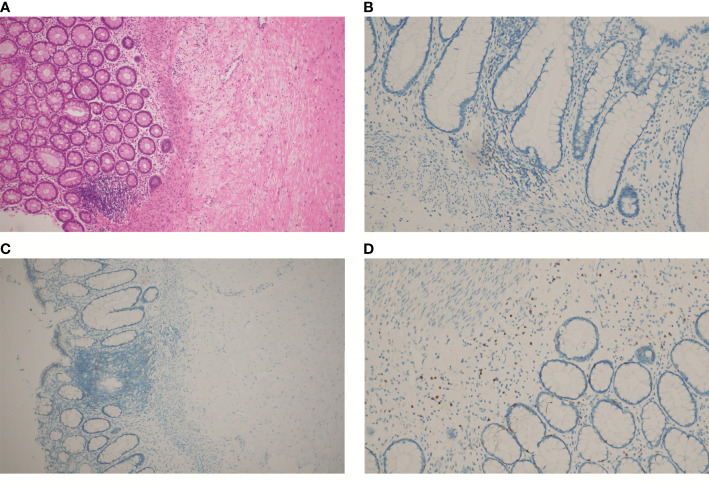
HE and IHC staining on tumor tissues of the patient after chemotherapy, immunotherapy, and surgery: **(A)** HE staining; **(B)** IHC staining of CD3; **(C)** IHC staining of PD-1; **(D)** IHC staining of CD8. **(A, C)**, at 100× magnification; **(B, D)**, at 200× magnification).

Due to alopecia and other toxic side effects, the patient refused to use irinotecan second-line therapy. Due to the cost, she refused BRAF V600E mutation drug combined with anti-EGFR monoclonal antibody targeted therapy. A trial of immunotherapy was requested. Two cycles of immunotherapy with Camrelizumab 200 mg + XELOX + Bevacizumab were completed from May 2021 to June 2021. However, she complained of concurrent nausea, vomiting, and myelosuppression. The regimen was changed to Camrelizumab + Regorafenib from July 2021 to August 2021 for two cycles after multi-disciplinary treatment (MDT). After the treatment, the CT review scan revealed that compared to the May CT film, the rectal wall and sigmoid colon are less thickened than before, and the peritoneal nodules disappeared. However, the patient developed oral ulcers and erythema on the hands and feet after taking Regorafenib, which was considered as hand–foot syndrome grade 2. The symptoms continued to worsen after dose reduction. From September 2021 to November 2021, she discontinued Regorafenib and received Camrelizumab + Cetuximab + Vemurafenib regimen. On 29/10/2021, the CT review scan suggested slight reduction in intestinal wall thickening compared with the slight reduction in rectal mesenteric fascia and presacral fascia thickening and little change in scattered multiple nodular shadows in the superior rectal artery travel area, presacral fascia, peritoneum, and omentum compared with the previous film. PET-CT suggested that no significant increase in metabolic activity was seen after rectal cancer treatment, and metabolic activity was suppressed after rectal cancer treatment. Colonoscopy showed that after neoadjuvant treatment for rectal cancer, only a few nodules remained after significant shrinkage of the tumor. On 16/11/2021, Vemurafenib treatment was suspended due to pancreatitis, and Cetuximab + Camrelizumab treatment continued. At 2021-12-01, the patient took oral Vemurafenib for CA19-9 elevation voluntarily, further triggering severe pancreatitis, and then Immunotherapy and targeted therapy were suspended. On 04/01/2022, the regimen was adjusted to Vemurafenib combined with Cetuximab and further adjusted to Trametinib + Dabrafenib + Cetuximab in February 2022. She eventually underwent anterior rectal resection in April 2022. The postoperative pathological biopsy found no signs of malignancy and only suggested chronic inflammation of the mucosa with focal lymphoid tissue reactive hyperplasia (**Figure 4A**). Meanwhile, immunohistochemistry (IHC) revealed tiny amounts of CD3 (+) (**Figure 4B**), scattered CD8 (+) (**Figure 4D**), and minor amounts of PD-1 (+) (**Figure 4C**).

## Discussion

CRC ranks third in having the highest incidence rate and second in having the highest mortality rate worldwide (1). With the continuous development of anti-cancer drugs, CRC patients are being treated more and more effectively. However, its good prognosis still relies heavily on the implementation of surgery (2, 3). The treatment of patients with unresectable mCRC remains a pain point for surgeons all over the world, especially for those with MSS and BRAF V600E mutation, who has been reported to have the worst prognosis (6).

Immunotherapy has been a hot topic in cancer treatment in recent years, including CRC. Previous studies have found that tumor cells could express PD-1 ligands PDL1 or PDL2. Overexpressed PD-L secreted from tumor cells could bind to PD-1 receptors in immune cells such as T cells and B cells, thereby inhibiting their function to achieve immune escape. The effect of immunotherapy is to inhibit the expression of PD-1 receptors in immune cells and expose tumor cells to immune cells (8–10).

Nowadays, Encorafenib and Cetuximab combined with Nivolumab were found to be effective in patients with MSS, BRAF V600E-mutated CRC (7). However, none of these patients achieved complete remission. In this case report, the patient had unresectable extensive peritoneal metastases from MSS BRAF V600E-mutated rectal cancer. She achieved pathological complete remission after being treated with Vemurafenib and Cetuximab in combination with Camrelizumab.

As a regulator of the MAPK/ERK signaling pathway, the BRAF inhibitor could disrupt negative feedbacks, which could lead to an induction of RAS activity and activation of other RAF kinases to bypass the effects of the BRAF inhibitor (11–15). The BRAF V600E-mutant mCRC had a poor response to selective BRAF inhibitor such as Vemurafenib. While the epidermal growth factor receptor (EGFR) might play a pivotal role in the increased RAS activity following BRAF inhibition, here, we combined BRAF and EGFR inhibition to produce improved MAPK suppression, which was approved by the United States Food and Drug Administration (US FDA) in April 2020 (BRAFTOVI, Array BioPharma Inc.). Finally, the BRAF V600E inhibitor Vemurafenib and EGFR antibody Cetuximab in combination with PD-1 inhibitor Camrelizumab were confirmed as the treatment regimen after multidisciplinary discussion (MDT) discussion.

Despite further progression after the initial use of the anti-VEGF drug bevacizumab in combination with concurrent radiation therapy, we suggested that they played an important role in this successful treatment. It was universally acknowledged that anti-VEGF drugs could inhibit tumor angiogenesis. The hypoxia and elevated interstitial fluid pressure in the tumor microenvironment (TME) caused by this promoted the entry of a great number of immune cells into the tumor tissue, which was supported by the IHC staining of marginally positive CD3, scattered positive CD8, and scattered positive PD1. Additionally, anti-VEGF drugs prevented the reduction in adhesion molecule expression to facilitate immune cells’ access to tumor tissue (10, 16). Meanwhile, it has been demonstrated that radiotherapy might induce endoplasmic reticulum stress to make tumor cells more easily recognized by CTL, in parallel with direct tumor cell killing (17).

In addition, Aaron M. Goodman et al. reported that high TMB was an independent predictor for the response to immunotherapy in diverse cancers (18), and it was confirmed by meta-analysis that TMB can be used as a potential predictive biomarker of CRC patients receiving immunotherapy (19). However, the definition of high TMB was unclear. Federico Innocenti et al. defined 8 or more mutations/Mb as high TMB, and patients with high TMB had a better overall survival (OS) regardless of the first-line treatment (20). Here, the patient had a high TMB (9.75 mutations/Mb), indicating a high number of tumor-associated neoantigens, which could favor the identification of cancer cells by the host immune system to achieve final pathological complete remission as well.

In conclusion, this case suggested that anti-VEGF drug Vemurafenib and Cetuximab in combination with PD-1 inhibitor Camrelizumab were definitely effective in MSS, BRAF V600E-mutated mCRC. To benefit more patients, further studies need to be completed.

## Author contributions

Agreeing to be accountable for all aspects of the work, all authors participated in perioperative data collection, manuscript drafting or revising, and decision making to which journal the manuscript would be submitted.
