# Supplementary figures and images for: Does PD-1 blockade play a decisive role in the pathological complete remission of unresectable MSS, BRAF V600E-mutated metastatic colorectal cancer: A case report
Source: Front Oncol. 2023 Jan 13;12:976622. doi: 10.3389/fonc.2022.976622 (PMC9880525; doi:10.3389/fonc.2022.976622)

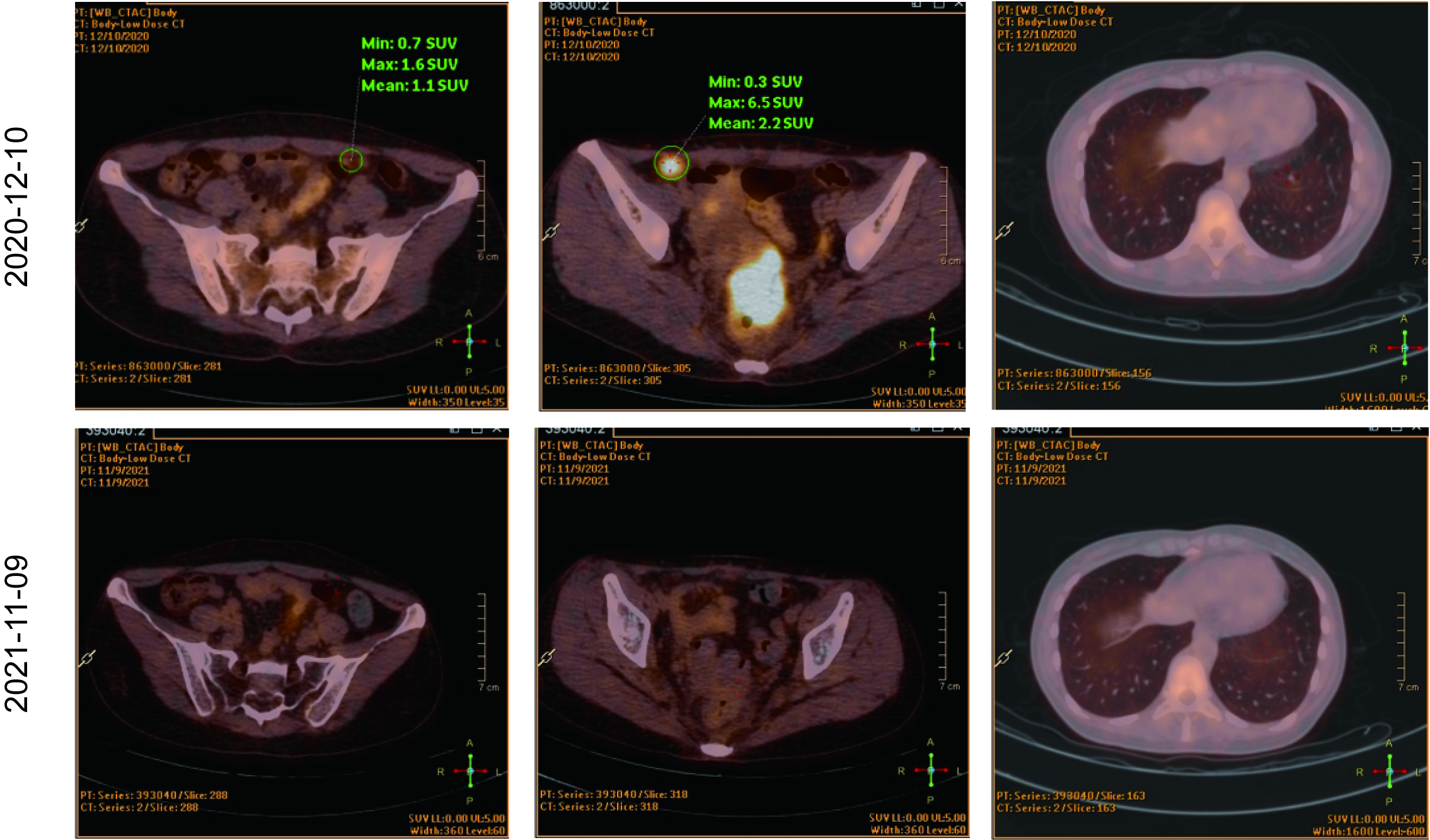

Supplement: Supplementary file 1 [file Image_1.tif]

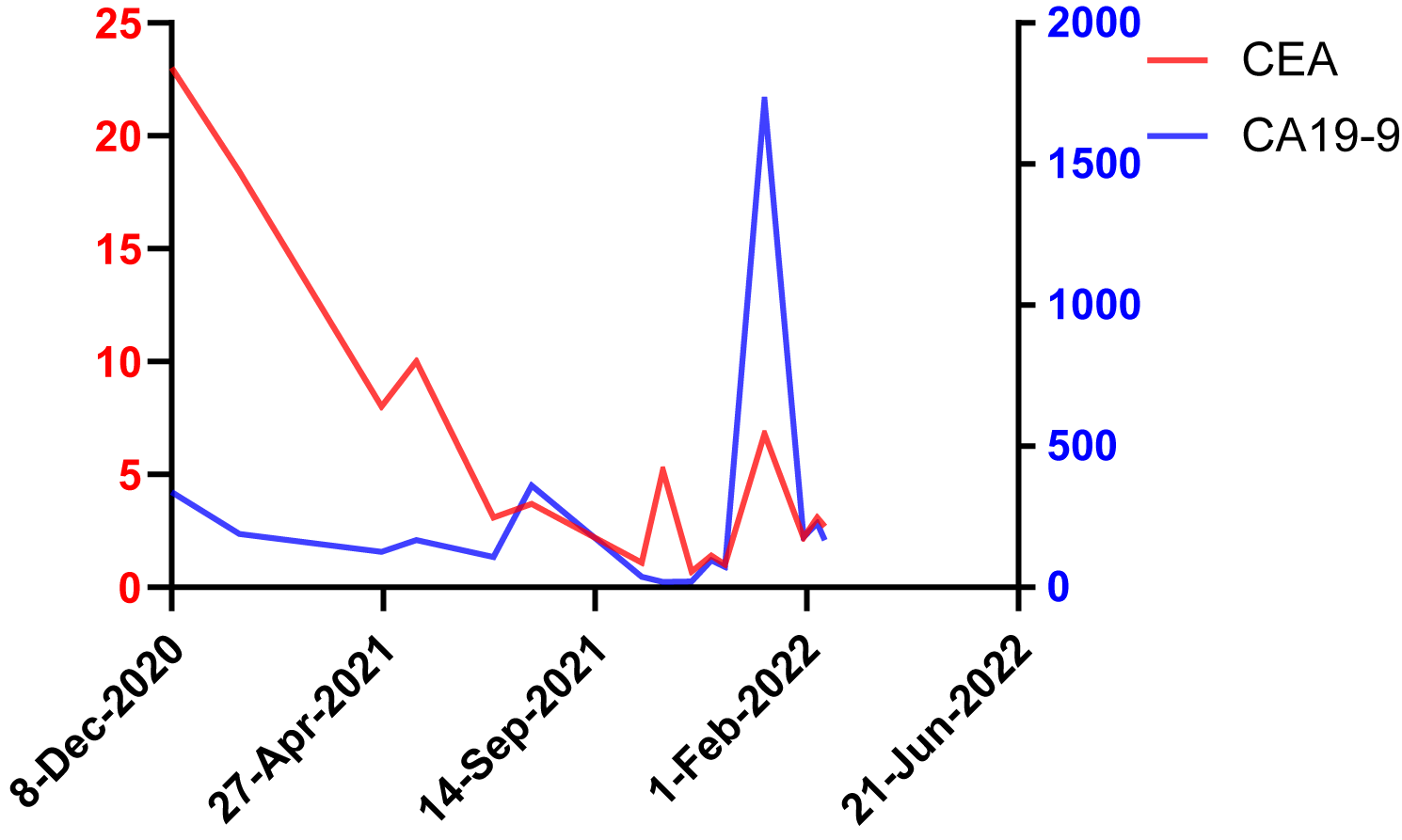

Supplement: Supplementary file 2 [file Image_2.tif]

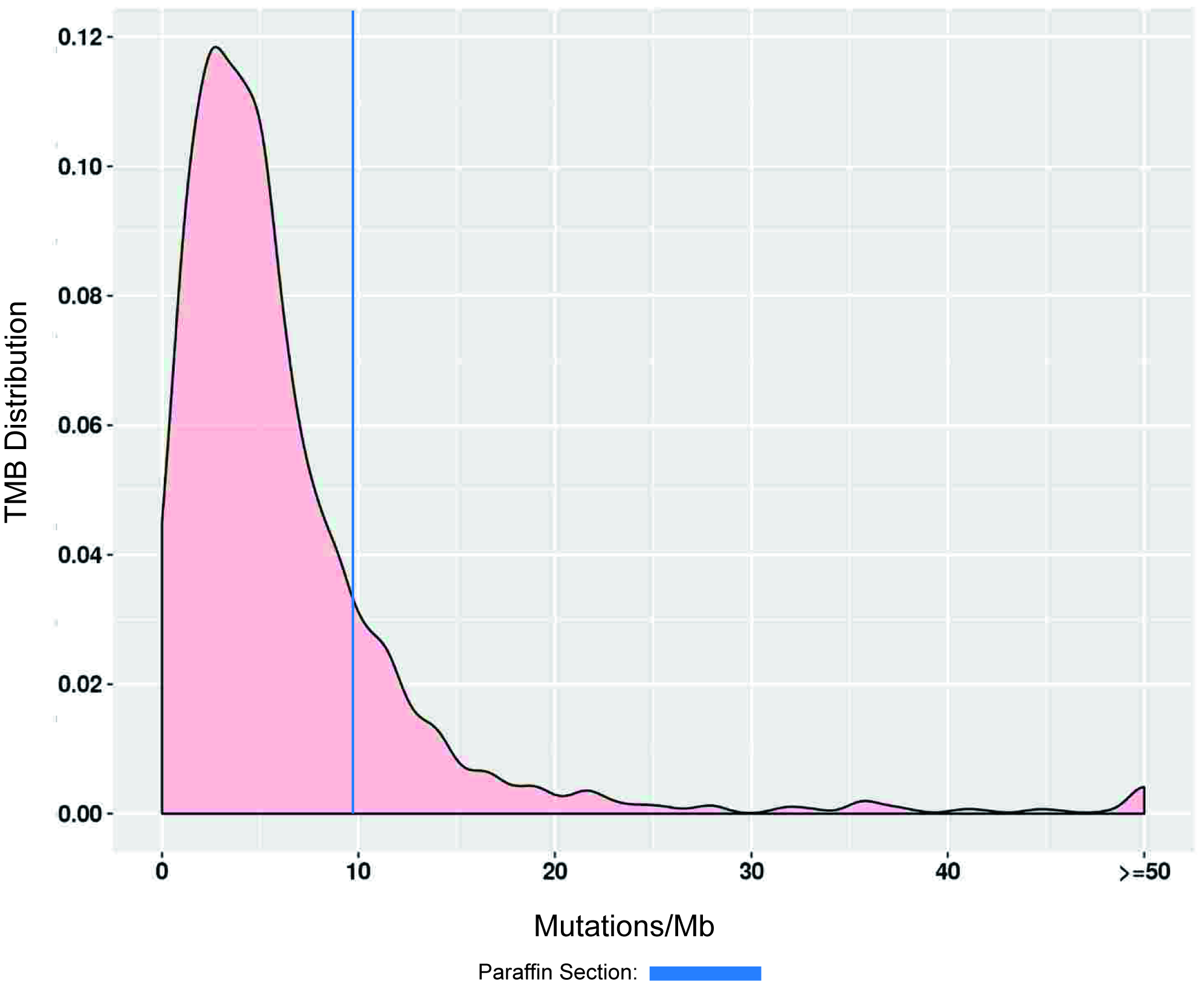

Supplement: Supplementary file 3 [file Image_3.tif]
